# Enhanced Bioaccessibility of Crocetin Sugar Esters from Saffron in Infusions Rich in Natural Phenolic Antioxidants

**DOI:** 10.3390/molecules201017760

**Published:** 2015-09-25

**Authors:** Stella A. Ordoudi, Anastasia Kyriakoudi, Maria Z. Tsimidou

**Affiliations:** Laboratory of Food Chemistry and Technology, School of Chemistry, Aristotle University of Thessaloniki, Thessaloniki 54124, Greece; E-Mails: steord@chem.auth.gr (S.A.O.); ankyria@chem.auth.gr (A.K.)

**Keywords:** crocetin sugar esters, saffron infusions, phenolic antioxidants, *in vitro* digestion, bioaccessibility

## Abstract

The present study aims to examine whether and to what extent the bioaccessibility of the major saffron apocarotenoids, namely crocetin sugar esters (CRTSEs), is affected by the presence of strong water-soluble antioxidants, ingredients of the herbs found in commercial tea blends with saffron. An *in vitro* digestion model was applied to infusions from these products to investigate the possible changes. All of the studied infusions were rich in total phenols (9.9–22.5 mg caffeic acid equivalents/100 mg dry infusion) and presented strong DPPH radical scavenging activity regardless of the composition of the corresponding herbal blends. RP-HPLC-DAD and LC-MS analysis enabled the grouping of the infusions into hydroxycinnamic acid-rich and in flavan-3-ol-rich ones. CRTSEs in herbal tea infusions were found to be significantly more bioaccessible (66.3%–88.6%) than those in the reference saffron infusion (60.9%). The positive role of strong phenolic antioxidants (caffeic acid, rosmarinic acid) on the stability of CRTSEs was also evidenced in model binary mixtures. On the contrary, cinnamic acid, exerting no antioxidant activity, did not have such an effect. Our findings suggest that strong radical scavengers may protect the crocetin sugar esters from oxidation during digestion when present in excess.

## 1. Introduction

Crocetin esters with sugars (C_20_H_24_O_4_, 8,8′-diapocarotene-8,8′-dioic acid) (CRTSEs) selectively known as crocins, are a group of water-soluble apocarotenoids rarely found in nature. The dried red stigmas of *Crocus sativus* L. that comprise the most expensive spice in the world, saffron, are currently the only edible source of these esters. Although the content of CRTSEs is affected by processing and storage conditions, these secondary metabolites account for *ca*. 30% of the spice weight soon after its processing [1]. Saffron can be considered as a functional spice [2] because of the many biological activities assigned to its extracts or individual apocarotenoids, after oral or intraperitoneal administration in doses higher than those daily consumed through food seasoning. It is worth mentioning that although substances intravenously administered are considered to be fully bioavailable, this is not the case for the orally-obtained ones. Therefore, the knowledge about the bioaccessibility of crocetin sugar esters, *i.e.*, the amount that is available for absorption in the gut after digestion [3], is of particular interest for future saffron intake recommendations and for the development of new products. The first published data [4] indicated that ~50% of the CRTSEs from aqueous saffron extracts are bioaccessible under *in vitro* digestion conditions. However, there is no information so far about the bioaccessibility of these compounds in food preparations to which saffron is added at quantities higher than those needed to impart its coloring and flavor properties. Herbal tea blends with saffron comprise such a category in the market of functional foods and beverages.

The bioaccessibility of a particular compound depends, often heavily, on interactions with other dietary constituents [5]. This issue is now gaining interest among scientists, who work on the interactions of compounds in complex matrices. Characteristic examples are the studies on the bioaccessibility of phenolic compounds and carotenoids in blended fruit-juice containing orange, kiwi, pineapple and mango [6] and on the effect of milk on the bioaccessibility of the phenolic compounds of black tea [7]. In this view, the present study aims to examine whether and to what extent the bioaccessibility of CRTSEs is affected by the presence of strong water-soluble antioxidants, ingredients of the herbs found in commercial tea blends with saffron.

Teas (from the dried leaves of *Camellia sinensis*) or herbal teas (from leaves, flowers, roots, *etc.*, of other plant species) are among the most widely-consumed beverages across the world. Such kinds of products are commercially available and attract the interest of consumers not only for their flavor, but also due to the biological activities assigned to certain ingredients. Epidemiological evidence associates the consumption of herbal teas with various health effects [8]. Some of them have been attributed to the antioxidant properties of endogenous phenolic compounds, such as flavanols, flavanones, as well as hydroxycinnamic and *p*-hydroxybenzoic acids, *etc.*

In the present study, the bioaccessibility of CRTSEs in infusions prepared from commercial herbal tea blends with saffron was examined using the same gastrointestinal model with that previously employed in our laboratory for comparison [4]. Chemical characterization of the infusions before and after digestion was used to investigate changes in the content of CRTSEs in the presence of phenolic antioxidants. Model binary mixtures of CRTSEs and selected phenolic compounds were also tested under the abovementioned digestion conditions to provide further support to the findings so far.

## 2. Results and Discussion

### 2.1. Extractability and Stability of CRTSEs under the Infusion Preparation Conditions

Infusion of a tea bag containing only saffron and no other herb in hot water (90 °C) for five minutes resulted in almost 80% (*n* = 3) recovery of its total CRTSE content. The latter was determined under exhaustive extraction conditions by RP-HPLC-DAD analysis according to [1]. Complete recovery of CRTSEs (100%, *n* = 3) was achieved only when the infusion period was doubled (10 min). This finding indicated that soaking saffron in hot water for 5 min does not heavily affect the stability of its apocarotenoids, in line with data from past kinetic studies [9,10]. On the other hand, extraction with cold water was far less effective, as it resulted in only 27% recovery of CRTSEs within the suggested time for the preparation of infusions (5 min). Manufacturer’s instructions seemed, thus, to be appropriate for the quantitative release of these bioactive metabolites from their matrix to infusion and were adopted in the following experiments.

### 2.2. Changes in the Profile and Content of CRTSEs upon in Vitro Gastrointestinal Digestion

**Figure 1:**
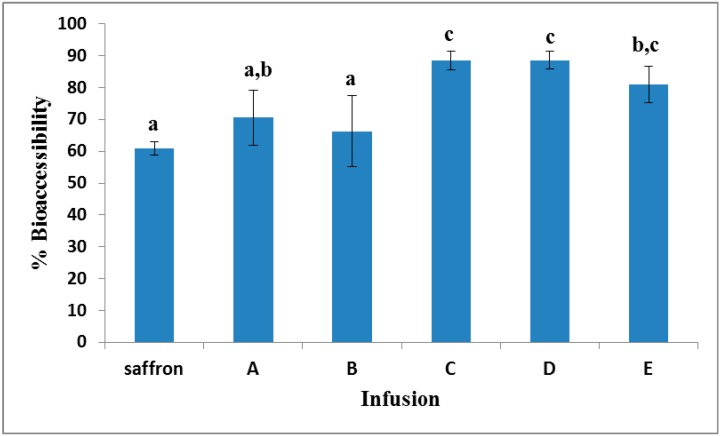
The % bioaccessibility of total crocetin sugar esters (CRTSEs) in infusions of saffron and herbal tea blends with saffron (A–E). Data are the mean values of three independent experiments. Different lowercase letters (a–c) indicate significant differences among infusions according to Duncan’s test (*p* < 0.05).

RP-HPLC-DAD analysis of non-digested infusions indicated that all of them had a similar content of crocetin sugar esters ($\bar{x}$ = 4.7 ± 1.1 mg/100 mg dry infusion, *n* = 5) with *trans*-crocetin di-(β-d-gentiobiosyl) ester (*trans*-4-GG crocetin ester) being the major one (>80% of the total CRTSE content). Aliquots of the prepared infusions were then directly subjected to simulated gastrointestinal digestion. The % bioaccessibility values for total crocetin sugar esters in Infusions A–E are shown in Figure 1 together with that found for a saffron infusion. The amount of saffron was the same as in the rest of herbal blends with saffron tea bags.

After the simulated digestion of the saffron infusion, the remaining amount of CRTSEs reached approximately 60% of the initially-determined one. This value was in line with the finding of our previous study [4] in which crocetin esters from an aqueous saffron extract (50 mg/L) were found to be bioaccessible by almost 55%. Losses can be attributed to the combined effect of the acidic and basic pH of the gastric and small intestinal phases of digestion, respectively, as well as to the physiological temperature of the human body (37 °C), rather than to the presence of digestive enzymes (pepsin, pancreatin) and bile salts. Figure 1 illustrates that Infusions A and B presented a rather similar bioaccessibility compared to that of the saffron infusion (70.5% and 66.3%, respectively). The % bioaccessibility of total CRTSEs was significantly higher in Infusions C, D and E (88.5%, 88.6% and 80.9%, respectively), and this result could not be considered as a random one. Positive interactions between dietary constituents that lead to an increase in the bioaccessibility of precious ingredients have been reported in the literature. For example, Podsędek and co-workers [11] observed that anthocyanins were more bioaccessible in the presence of other red cabbage constituents than when digested *per se*. A similar hint was given in our previous work, when the difference in the bioaccessibility of CRTSEs between saffron and gardenia aqueous extracts was evidenced [4]. Taking into account the list of ingredients of the blends on the labeling and the relevant literature, the infusions are expected to be rich in various classes of phenolic compounds with strong, moderate or weak antioxidant activities. Yet, it is unknown whether these constituents protect crocetin esters against oxidation reactions that take place upon gastrointestinal conditions, as has been reported for other oxidizable constituents [12].

### 2.3. Total Phenol Content and DPPH Radical Scavenging Activity of Infusions

**Figure 2:**
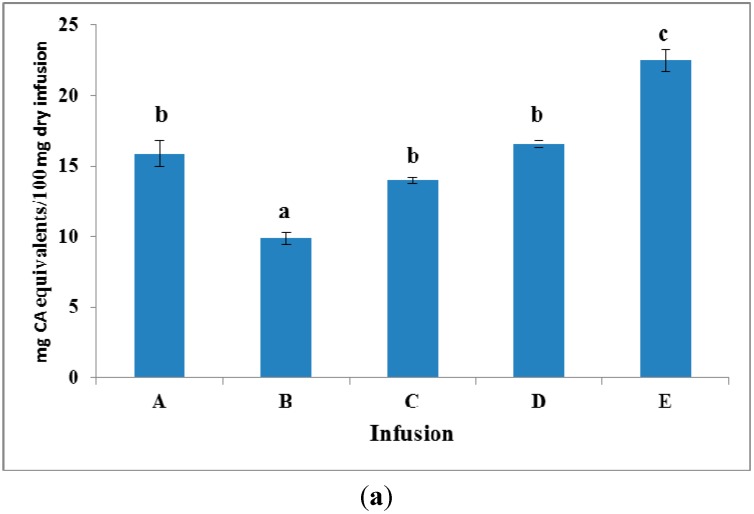

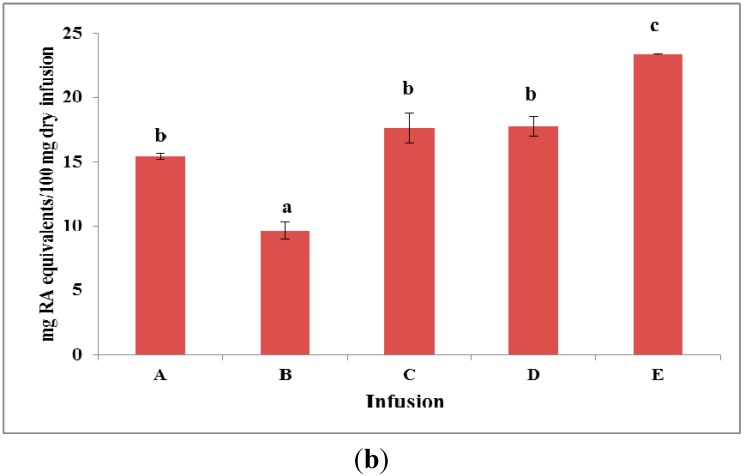
(**a**) Total phenol content (expressed as caffeic acid equivalents, CA) and (**b**) DPPH radical scavenging activity (expressed as rosmarinic acid equivalents, RA) of Infusions A–E. Data are the means of three independent measurements ± SD; different lowercase letters (a–c) indicate significant differences among infusions according to Duncan’s test (*p* < 0.05).

Under the chromatographic conditions for the separation of CRTSEs, many peaks were also detected in the UV region with maxima at 270 and 330 nm, indicating the presence of constituents containing phenolic moieties with antioxidant properties. The total phenol content and the radical scavenging activity of infusions were then estimated, and the results shown in Figure 2a,b verified the abovementioned evidence.

Figure 2a shows that the total phenol content of the examined infusions, as determined by the F–C assay, varied from 9.9–22.5 mg caffeic acid equivalents/100 mg dry infusion. The highest total phenol content was observed for Infusion E followed by those of the D and A blends that are labeled to contain mainly green, black and rooibos tea, respectively. All of the three mentioned herbs are well-established sources of polyphenols. For example, *Camellia sinensis* leaves (green and black tea) contain high amounts of flavan-3-ols (*i.e.*, catechin, epicatechin derivatives) [13], whereas *Aspalathus linearis* leaves (rooibos tea) are rich in dihydrochalcones (aspalathin) and flavones (orientin) [14]. The lowest content of total phenols was observed in Infusion B, containing spearmint and orange tree leaves as the major herbs. In that case, small molecular mass phenols belonging to hydroxycinnamic acids (e.g., rosmarinic acid) are expected to predominate [15,16]. It should be noted that the latter infusion was also the one with the lowest bioaccessibility value for CRTSEs. Moreover, infusions from the C and D blends that presented the highest bioaccessibility for CRTSEs differed only slightly in their total phenol content originating from different kinds of herbs; peppermint and black tea were claimed on the respective labels to be the major herbal tea ingredients (>50%). Infusions from *Mentha × piperita* L. (peppermint) are rich in flavanone and flavone glycosides, as well as hydroxycinnamic acids, e.g., [17], but do not seem to contain any flavan-3-ols. The latter are found in abundance in black tea. The DPPH radical scavenging activity of the studied infusions (Figure 2b) showed a similar trend to that evidenced for the total phenol content. Infusion E was found to be the richest in strong radical scavengers (23.4 ± 0.1 mg RA equivalents/100 mg dry infusion) followed by D, C and A, which were practically equipotent (17.8 ± 0.8, 17.7 ± 1.2, 15.4 ± 0.2 mg RA equivalents/100 mg dry infusion, respectively). Infusion B, being the poorest in total phenol content, exerted also the lowest antiradical activity (9.7 ± 0.7 mg RA equivalents/100 mg dry infusion). Considering that aqueous extracts of saffron are not expected to contain appreciable amounts of phenolic compounds or to exhibit *in vitro* radical scavenging activity [4,18], the observed values of antiradical activity were attributed to the co-presence of the phenolic ingredients of the herbs.

Overall, the infusions were found to contain phenolic compounds at considerably higher levels (2–4-fold) in comparison to that of crocetin sugar esters. In order to examine whether the existing phenolic antioxidants positively affect the stability of CRTSEs, the bioaccessibility of the latter was tested in a model system containing approximately the mean amount of apocarotenoids recovered in the infusions (0.4 mg/100 mg dry infusion) and an excess of rosmarinic acid, a well-known potent radical scavenger, at ratio values of 1:3, 1:6 and 1:12 (*w*/*w*). The reference sample contained only crocetin esters at the same concentration. The solutions were subjected to digestion, and the % bioaccessibility of crocetin esters was determined (Table 1). Results indicate a clear protective effect of rosmarinic acid when present in excess. This means that when the content of rosmarinic acid exceeded at least three-fold that of CRTSEs, a positive, but not concentration-dependent effect on the stability of the latter compounds was observed.

The % bioaccessibility of crocetin esters was higher than the obtained value for the reference sample by 17–18 percentage units. On the other hand, the % bioaccessibility values of rosmarinic acid in the mixture with crocetin esters were 6%–10% lower than those found when it was examined alone at the three studied levels ($\bar{x}$ = 97.4% ± 2.3, *n* = 3). This finding could be associated with the fact that rosmarinic acid is partially spent in order to protect the oxidizable crocetin esters during the *in vitro* digestion.

**Table 1:** The % bioaccessibility of crocetin sugar esters (CRTSEs) and rosmarinic acid (RA) in model mixtures upon simulated gastrointestinal conditions.

CRTSEs: Rosmarinic Αcid (*w*/*w*)	% Bioaccessibility ^a,b^	
	CRTSEs	Rosmarinic Αcid
1:12	79.6 ± 6.7 ^b^	89.6 ± 5.1 ^a^
1:6	76.9 ± 4.1 ^b^	88.8 ± 2.3 ^a^
1:3	77.7 ± 4.0 ^b^	84.8 ± 3.3 ^a^
1:0	60.1 ± 4.9 ^a^	-

^a^ Values are expressed as the mean ± SD; ^b^ different lower case letters within the same column indicate significant differences according to Duncan’s test (*p* < 0.05).

To investigate whether there is a relationship between the structure of the phenolic compound and its protective effect on CRTSEs during the digestion, two other structurally-related cinnamic acids, namely caffeic acid and cinnamic acid, were studied in model solutions. The two acids were used at a level equimolar to that of rosmarinic acid when present in a six-fold higher concentration than that of CRTSEs. Caffeic acid is a well-known strong phenolic antioxidant due to its ortho-dihydroxy moiety [19], while cinnamic acid, lacking hydroxyl groups, can be considered as a blind test compound. The % bioaccessibility of crocetin esters was compared to the values obtained for the reference sample (Table 2). The results show that caffeic acid and rosmarinic acid brought about a significant enhancement in the % bioaccessibility of CRTSEs; almost 36% and 27% higher values than those evidenced in the reference sample. No positive effect on the bioaccessibility of CRTSEs was observed in the presence of cinnamic acid, which does not bear a phenolic moiety. This finding signifies that the phenolic compounds may protect the CRTSEs during digestion due to their radical scavenging properties.

**Table 2:** The % bioaccessibility of CRTSEs and of selected phenolic compounds upon simulated gastrointestinal conditions.

Model Solution Composition	Bioaccessibility ^a,b^	
	% Crocetin Esters	% Phenolic Compound
CRTSEs ^c^:caffeic acid ^d^	87.6 ± 5.5 ^c^	95.2 ± 3.6
CRTSEs ^c^:cinnamic acid ^d^	49.5 ± 1.9 ^a^	89.3 ± 6.8
CRTSEs ^c^:rosmarinic acid ^d^	78.7 ± 1.0 ^b^	84.9 ± 4.5
Reference sample	51.6 ± 1.5 ^a^	-

^a^ Values are expressed as the mean ± SD; ^b^ different lowercase letters (a–c) within the same column indicate significant differences according to Duncan’s test (*p* < 0.05); ^c^ 4.7 µM as *trans*-4-GG crocetin ester; ^d^ 85 µM of rosmarinic acid, caffeic acid or cinnamic acid.

### 2.4. Changes in the Phenolic Profile and Individual Phenol Content of Infusions A–E upon Gastrointestinal Digestion

The composition of the examined infusions regarding their phenol content is expected to be complex considering that different herbs were used for their preparation. Based on available standards, UV-VIS spectra, mass spectra and literature data on the phenolic composition of the herbs, the examined infusions were divided into two groups; those rich in hydroxycinnamic acids (A, B and C) and those rich in flavan-3-ols (D and E). In Table 3, quantitative data for the concentration of total and major individual phenolic compounds of each class before and after digestion, as well as the respective % bioaccessibility values are given. Observations regarding the major class of phenolic compounds present in each infusion are given below.

**Table 3:** Contents of total hydroxycinnamic acids and rosmarinic acid (330 nm) of Infusions A–C, as well as flavan-3-ols (270 nm) and (−)-epicatechin of infusions D and E (mg/100 mg dry infusion) before and after simulated digestion.

Infusion	Before Digestion	After Digestion	% Bioaccessibility ^a^
mg/100 mg dry infusion ^a,b^			
	Total hydroxycinnamic acids ^c^/rosmarinic acid (330 nm)		
A	3.09 ± 0.02 ^a^/1.53 ± 0.04 ^a^	2.71 ± 0.14 ^a^/1.09 ± 0.03 ^b^	87.6 ± 4.1/71.4 ± 2.9
B	2.70 ± 0.01 ^a^/1.92 ± 0.03 ^a^	2.12 ± 0.30 ^b^/1.76 ± 0.07 ^a^	78.6 ± 10.8/91.9 ± 3.2
C	2.44 ± 0.06 ^a^/1.57 ± 0.04 ^a^	2.09 ± 0.41 ^b^/1.71 ± 0.02 ^a^	86.2 ± 19.1/108.7 ± 3.9
	Total flavan-3-ols ^d^/(−)-epicatechin (270 nm)		
D	12.99 ± 0.27 ^a^/12.23 ± 0.30 ^a^	12.91 ± 0.60 ^a^/10.68 ± 0.38 ^a^	99.3 ± 2.2/87.3 ± 1.3
E	19.47 ± 0.60 ^a^/13.89 ± 0.26 ^a^	18.35 ± 0.30 ^a^/12.60 ± 0.16 ^a^	94.3 ± 2.9/90.8 ± 1.5

^a^ Values are expressed as the mean ± SD; ^b^ different lower case letters within the same row for each infusion tested before and after digestion indicate significant differences according to Duncan’s test at *p* < 0.05; ^c^ expressed as rosmarinic acid equivalents; ^d^ expressed as (−)-epicatechin equivalents.

#### 2.4.1. Changes Observed in the Class of Hydroxycinnamic Acids

**Figure 3:**
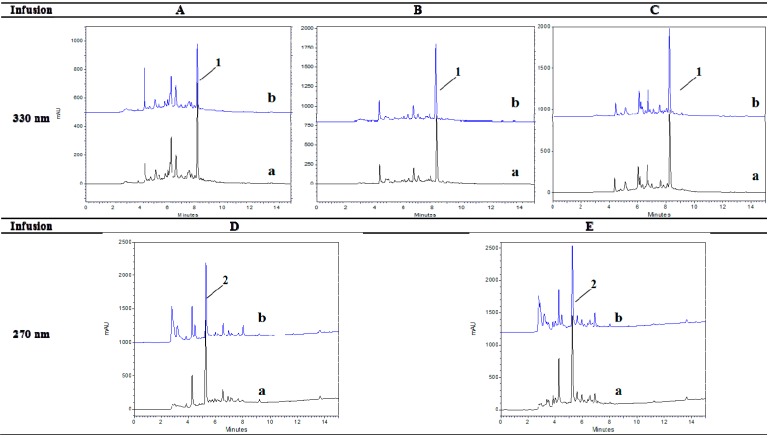
RP-HPLC-DAD profile of Infusions (**A**–**C**) at 330 nm and of Infusions (**D**) and (**E**) at 270 nm before (a) and after (b) simulated digestion. Peak assignment: Peak 1, rosmarinic acid; Peak 2, (+)-catechin. Chromatographic conditions were as described in the Experimental Section.

Infusions A, B and C that contained several herbs of the *Lamiaceae* family (sage, rosemary and mint, respectively) were found to be rich in compounds that bear hydroxycinnamic acid moieties. The latter were quite stable under the gastrointestinal digestion conditions with % bioaccessibility values higher than 78% (Table 3). Increased stability has been also reported for hydroxycinnamic acids in the case of wines following gastric and intestinal treatment [20]. Taking into account that constituents of this class of phenolic compounds were similarly stable in the three infusions under digestion conditions, the observed differences in the % bioaccessibility of CRTSEs from Infusions A, B and C could not be easily explained. In this view, attention was paid to the fate of major individual compounds upon the gastrointestinal digestion conditions. Rosmarinic acid (*t*_R_ = 8.20 min, λ_max_ = 293/329 nm, *m*/*z* = 359) (Figure 3, Peak 1) was found to be the predominant constituent in Infusions A–C (1.53–1.92 mg/100 mg dry infusion), as expected [21]. Its content exceeded that of CRTSEs in all three infusions by 2–6 times, not only before, but also after digestion. In line with what was evidenced in the model system, this finding indicates once more that rosmarinic acid in excess may be beneficial for the stability of CRTSEs during digestion. Still, it is not possible to estimate the exact concentration of this phenolic compound, or total hydroxycinnamic acids that becomes critical for the bioaccessibility of CRTSEs.

Rosmarinic acid was found to be rather stable upon the simulated gastrointestinal digestion applied to Infusions A–C with % bioaccessibility values presenting greater variation (71%–109%) than that found for total hydroxycinnamic acids among infusions (Table 3). Such variation could be attributed to the fact that the stability of phenolic compounds can be influenced by interactions among them or with other constituents in a synergistic or antagonistic way [22]. The chromatographic profiles at 330 nm (Figure 3) clearly illustrate the quantitative changes in the content of rosmarinic acid in Infusions A–C before and after digestion. In particular, the rosmarinic acid concentration increased after digestion (% bioaccessibility > 100%) in Infusion C. This could be due to the presence of complex structures in the infusions that could be broken down by acidic conditions or digestive enzyme action to liberate rosmarinic acid monomers. The same observations have been also made in the case of other phenolic compounds, such as the liberation of gallic acid monomers from galloyl-proanthocyanidins in wines [20]. In addition, Frontela and co-workers [23] have reported a significant increase in the concentration of certain phenolic acids after the *in vitro* digestion of several fruit juices.

#### 2.4.2. Changes Observed in the Class of Flavan-3-ols

Only Infusions D and E that contained black and green tea as the major ingredients (>50%) were found to be rich in compounds with flavan-3-ol structure. Total flavan-3-ols were found to be highly bioaccessible (>94%) (Table 3). However, qualitative differences in the chromatographic profiles of Infusions D and E before and after digestion were evidenced at 270 nm (Figure 3). Certain unidentified peaks at *t*_R_ = 2.9 min, 3.2 min, 4.5 min and 7.7 min in Infusion D and at *t*_R_ = 2.9 min, 3.2 min, 4.5 min and 6.9 min in Infusion E were increased after digestion. This could be due to the liberation of monomers from complex chemical structures under digestion conditions, as previously mentioned. In these infusions, (+)-catechin (*t*_R_ = 5.24 min, λ_max_ 238/278 nm, *m*/*z* 289) (Figure 3, Peak 2) was identified as the predominant constituent of this class before and after digestion. Its concentration exceeded that of CRTSEs by 43 times in D and 30 times in E. The bioaccessibility of catechin itself was found to be somewhat lower (<90%) than that of total flavan-3-ols, indicating that a part of its initial concentration could have been spent to protect CRTSEs. It should be stressed that CRTSEs from Infusions D and E were highly bioaccessible, as previously discussed (Figure 1).

## 3. Experimental Section

### 3.1. Samples

Commercially available herbal tea blends with saffron (A–E) were purchased from the market (Thessaloniki, Greece). Tea bags that contained only saffron at the same concentration as that claimed on the label of products were also prepared for comparison reasons. The saffron sample used as a control was of the same batch as that used in the particular lots of the commercial samples and was donated upon request by the manufacturer. Examination of the purity and quality of this saffron sample in the laboratory indicated that it was of the highest quality category according to the ISO 3632-1 specifications [24].

### 3.2. Standards, Reagents and Solvents

*Trans*-4-GG crocetin ester was laboratory isolated by semi-preparative reversed-phase high performance liquid chromatography [RP-HPLC, Marathon IV series HPLC pumps (Rigas Labs, Thessaloniki, Greece) coupled with a diode array linear UVVis-206 multiple wavelength detector (Linear Instruments, Fermont, California, CA, USA)] as previously described in detail by Kyriakoudi and Tsimidou [25]. The purity of isolated *trans*-4-GG (97%) was checked (1) chromatographically by RP-HPLC-DAD [pump model P4000 (Thermo Separation Products, San Jose, California, CA, USA), Midas autosampler (Spark, Emmen, The Netherlands) coupled with a UV 6000 LP diode array detector (DAD, Thermo Separation Products)] in the range of 200–550 nm and calculated as the percentage of the total peak area at 440 and (2) by nuclear magnetic resonance (NMR) spectroscopy recording the ^1^H 1D spectra at 300 MHz on a Brucker 300AM spectrometer (Bruker, Rheinstetten, Germany).

Caffeic acid (CA) (>98%) was from Sigma Chemical Co. (St. Louis, MO, USA). Rosmarinic acid (RA) (>95%) was from Fluka (Steinheim, Germany). Cinnamic acid (99%) was from Aldrich Chemical Cο. (Steinheim, Germany). (−)-Epicatechin was purchased from Biochemika (Duisburg, Germany). All of the chemicals from various suppliers were of the highest purity needed for each assay. In particular, the 1,1-diphenyl-2-picrylhydrazyl radical (DPPH·) was from Sigma Chemical Co. Folin-Ciocalteu (F-C) phenol reagent was obtained from Panreac Quimica (Barcelona, Spain). The digestive enzymes (pepsin, pancreatin) and bile salts used were from Sigma Chemical Co.

The acetonitrile, methanol (Chem-Lab, Zedelgen, Belgium) and acetic acid (Fluka Chemie, Buchs, Switzerland) used were of HPLC grade. Ultra-high purity water was produced using a SG Ultra Clear Basic UV system (SG Wasseraufbereitung und Regenerierstation GmbH, Barsbüttel, Germany).

### 3.3. Preparation of the Infusions

The preparation of the infusions was carried out according to the instructions of the manufacturer. In particular, one tea bag (1.8 g) was immersed in 400 mL of boiling water (95 °C) for 5 min. For the needs of the research, deionized tap water was used. For each type of blend (*n* = 5), ten infusions were prepared. Aliquots of 100 mL (*n* = 10) were then combined to form a representative sample that was used in all further analyses. A portion of the latter (50 mL) was lyophilized, and the rest was used as such. Samples were stored at −18 °C until analysis. Exhaustive extraction was assisted by an ultrasonic probe (duty cycles, 0.2 s; frequency, 20 kHz ± 500 Hz; output,70 W; amplitude, 100%) using a mixture of methanol:water (1:1, *v*/*v*) for 30 min [1].

### 3.4. Total Phenol Content by the Folin-Ciocalteu Assay

The total phenol content of the infusions was determined spectrophotometrically by the Folin-Ciocalteu (F-C) assay according to [19]. Caffeic acid (CA) was used as a reference standard, and results were expressed as mg CA equivalents/100 mg dry infusion on the basis of a calibration curve (y = 0.0099x − 0.0996, (10–100 μg/10 mL), *R*^2^ = 0.99 (*n* = 6)). Briefly, in a 10-mL volumetric flask, 5 mL of water, 0.4 mL of each representative sample and 0.5 mL F-C reagent were mixed. After exactly 3 min, 1.0 mL of saturated sodium carbonate solution (37%, *w*/*v*) was added, and the mixture was agitated. The volume was adjusted with water and the flask left in the dark for 1 h at room temperature. The absorbance was measured at 750 nm (U-2000 Hitachi UV-Vis spectrophotometer, Tokyo, Japan) against a blank prepared in the same way with deionized water in the place of the infusion aliquot. The repeatability of measurements calculated for a standard solution and an extract were found to be satisfactory (CV% = 2.0 and 3.0, respectively, *n* = 5). All of the subsequent measurements were then performed in triplicate for each sample, and results were expressed as the mean value.

### 3.5. DPPH Radical Scavenging Activity

The DPPH radical scavenging activity of representative samples was assessed according to the procedure presented by Nenadis and Tsimidou [26]. In brief, samples (0.05 mL) were added to 2.9 mL of a 0.1 mM methanolic solution of DPPH·. The absorbance at 515 nm was recorded at the start and after 30 min of incubation. A calibration curve of rosmarinic acid (RA) was prepared, and the results were expressed as mg RA equivalents/100 mg dry infusion. Measurements were carried out in triplicate. The repeatability of measurements calculated for a standard solution and an extract were found to be satisfactory (CV% = 3.0 and 4.3, respectively, *n* = 5).

### 3.6. In Vitro Gastrointestinal Digestion Procedure

Infusions prepared from various blends of herbs with saffron were used directly for the *in vitro* digestion procedure, which mimics the physiological conditions in the upper digestive tract (stomach and small intestine), according to [4]. Briefly, aliquots of the infusions were transferred into amber bottles, and Hank’s balanced salt solution (HBSS) was added to a final volume of 20 mL. To each bottle, 1 mL of freshly prepared pepsin (0.04 g pepsin/0.1 mol/L HCl) was added, and the pH was acidified to 2.0 using 1 mol/L HCl. The samples were overlaid with nitrogen gas and incubated at 37 °C for 1 h in a shaking water bath at 95 rpm to mimic the gastric phase of human digestion. The intestinal phase involved increasing the pH to 5.3 with 0.9 mol/L sodium bicarbonate followed by the addition of 200 μL of bile salts glycodeoxycholate (0.8 mmol/L), taurodeoxycholate (0.45 mmol/L) and taurocholate (0.75 mmol/L) and 100 μL of porcine pancreatin (0.08 g/mL HBSS). The final pH was adjusted to 7.4 using 1 mol/L NaOH. Samples were overlaid with a layer of nitrogen gas and incubated for 2.5 h at 37 °C to mimic the duodenal phase of human digestion. After the intestinal phase, the digestate was centrifuged at 4100 g using an SL 16R centrifuge (Thermo Scientific, Massachusetts, MA, USA) for 15 min at 4 °C, and the supernatants were collected and filtered through a 0.45-μm membrane filter (Sartorius Stedim Biotech GmbH, Goettingen, Germany) and stored at −18 °C until further analysis. The bioaccessibility of CRTSEs was calculated using Equation (1).

(1) $$\text{Bioaccessibility (\%) = }(\frac{C_{digested}}{C_{undigested}})\times 100$$

where *C_digested_* is the total CRTSEs concentration determined by HPLC after digestion and *C_undigested_* total CRTSE concentration before digestion.

The same gastrointestinal conditions were applied to model solutions containing CRTSEs and phenolic compounds at various ratios (see the Results and Discussion, Section 2.3.).

### 3.7. Liquid Chromatographic Analysis

#### 3.7.1. Crocetin Sugar Esters

High performance liquid chromatography (HPLC) was used to identify and quantify the crocetin sugar esters present in all of the infusions before and after the simulated *in vitro* digestion procedure. The HPLC system consisted of a pump, Model P4000 (Thermo Separation Products), a Midas autosampler (Spark) and a UV 6000 LP diode array detector (DAD) (Thermo Separation Products). Separation was carried out on a Discovery HS C18 (250 × 4.6 mm i.d.; 5 μm) column (Supelco, Bellefonte, USA). The elution system used consisted of a mixture of water:acetic acid (1%, *v*/*v*) (A) and acetonitrile (B). The linear gradient was 20%–100% B in 20 min. The flow rate was 0.8 mL/min. The injection volume was 20 μL. The analytical samples were prepared after proper dilution with deionized water (1:2, *v*/*v*) and filtration through a 0.45-μm membrane filter. Chromatographic data were processed using the ChromQuest Version 3.0 software (Thermo Separation Products). Monitoring was in the range of 200–550 nm. Peak identification was based on a comparison of retention times, UV-VIS spectra matching with those of available standards and literature data [27,28]. LC-ESI-MS (Model 2010 EV, Shimadzu, Kyoto, Japan) on positive and negative ion modes was also employed for the identification of the major crocetin esters. Separation was achieved on the same chromatographic column. Quantification of the total CRTSEs content (mg/100 mg dry infusion) was accomplished with the aid of a proper calibration curve of *trans*-4-GG crocetin ester.

#### 3.7.2. Phenolic Compounds

Analysis of phenolic compounds and peak identification was carried out under the same chromatographic conditions as those previously described for CRTSEs. Quantification in the UV-VIS region of individual groups of phenolic compounds, *i.e.*, hydroxycinnamic acids and flavan-3-ols, was carried out using proper calibration curves of rosmarinic acid (λ_max_ = 330 nm) and (−)-epicatechin (λ_max_ = 270 nm), respectively. The results were expressed as mg/100 mg dry infusion.

### 3.8. Statistical Analysis

Statistical comparisons of the mean values were performed by one-way analysis of variance (ANOVA), followed by the multiple comparison Duncan test (*p* < 0.05 confidence level) using the SPSS 14.0 software (SPSS Inc., Chicago, IL, USA).

## 4. Conclusions

Overall, our study shows for the first time that crocetin esters from saffron can interact synergistically with strong phenolic antioxidants during simulated gastrointestinal conditions so that they become more bioaccessible for absorption after oral intake. Taking into account that saffron apocarotenoids are considered bioactive at rather high daily oral doses, which cannot be attained through food seasoning, the findings of this study may be of particular interest for the functional food industry. In fact, the advantage of the enhanced bioaccessibility of crocetin esters from saffron in the presence of hydroxycinnamic acids or flavan-3-ols from other popular herbs can be further exploited in saffron bioactivity studies, future oral intake recommendations, as well as the design of new saffron-based functional beverages.
